# Exploring effective core drug patterns in primary insomnia treatment with Chinese herbal medicine: study protocol for a randomized controlled trial

**DOI:** 10.1186/1745-6215-14-61

**Published:** 2013-02-28

**Authors:** Shiyan Yan, Runshun Zhang, Xuezhong Zhou, Peng Li, Liyun He, Baoyan Liu

**Affiliations:** 1 Institute of Basic Research in Clinical Medicine, China Academy of Chinese Medical Sciences, No.16 NanXiaoJie, DongZhiMenNei, DongCheng District, Beijing, China; 2 Guang’anmen Hospital, China Academy of Chinese Medical Sciences, No.5 BeiXianGe St., XiCheng District, Beijing, China; 3 School of Computer and Information Technology, Beijing Jiaotong University, No.3 ShangYuanCun, Hai Dian District, Beijing, China; 4 China Academy of Chinese Medical Sciences, No.16 NanXiaoJie, DongZhiMenNei, DongCheng District, Beijing, China

**Keywords:** Primary insomnia, Chinese medicine, Randomized controlled trial, Effective core drug pattern

## Abstract

**Background:**

Chinese herbal medicine is one of the most popular Chinese medicine (CM) therapies for primary insomnia. One of the important characteristics of CM is that different Chinese clinicians give different prescriptions even for the same patient. However, there must be some fixed drug patterns in every clinician’s prescriptions. This study aims to screen the effective core drug patterns in primary insomnia treatment of three prestigious Chinese clinicians.

**Methods/design:**

A triple-blind, randomized, placebo-controlled, parallel-group clinical trial will be performed. Three clinicians will diagnose and treat every eligible patient individually and independently, producing three prescriptions from three clinicians for every patient. Patients will equally be randomized to one of four groups – medical group A, medical group B, medical group C, or placebo group – and observed for efficacy of treatment. The sample will include primary insomnia patients meeting DSM IV-TR criteria, Spiegel scale score >18, and age 18 to 65 years. A sequential design is employed. Interim analysis will be conducted when between 80 and 160 patients complete the study. The interim study could be stopped and treated as final if a statistically significant difference between treatment and placebo groups can be obtained and core effective drug patterns can be determined. Otherwise, the study continues until the maximum sample size reaches 300. Treatment of the CM group is one of three Chinese clinicians’ prescriptions, who provide independently prescriptions based on their own CM theory and the patient’s disease condition. Assessment will be by sleep diary and Pittsburgh sleep quality index, and CM symptoms and signs will be measured. Primary outcome is total sleep time. Assessment will be carried out at the washout period, weeks 1, 2, 3, and 4 and 4th week after the end of treatment. Effectiveness analysis will be per intent to treat. A multi-dimension association rule and scale-free networks method will be used to explore the effective core drug patterns.

**Discussion:**

The effective core drug patterns will be found through analyzing several prestigious CM clinicians’ treatment information. Screening the effective core drug patterns from prestigious clinicians can accelerate the development of new CM drugs.

**Trial registration:**

NCT01613183

## Background

Insomnia is a common public health problem. Approximately one-third to one-quarter of the population in developed nations has been reported to have sleep disturbance problems to some extent in their lives and approximately 10% suffer from persistent insomnia [1]. Population-based studies suggest that about 30% of the total population complains of sleep disruption, while approximately 10% has associated symptoms of daytime functional impairment with the diagnosis of insomnia, although it is unclear about the proportion among the 10% of population really suffering from chronic insomnia [2]. Pharmacologic therapy is the main treatment of insomnia. However, most of the drugs prescribed for insomnia have some risk of overdose, tolerance and addiction. Long-term use of frequently prescribed medications (for example, benzodiazepines) can lead to habituation and problematic withdrawal symptoms [3]. In China, most of patients prefer to receive traditional Chinese medicine (CM) treatment, including Chinese herbal medicine, acupuncture, massage and cupping [4-12]. In recent years, the traditional CM therapy for insomnia is enjoying increasing popularity in the West [5,6,13,14], reflecting growing acceptance of such treatments even in the West. Chinese herbal medicine is one of the most popular CM therapies for insomnia.

However, it is a common phenomenon that the efficacy of Chinese herbal drugs is related to the clinician. In other words, the efficacy of CM varies with different clinicians. Prestigious Chinese clinicians may therefore have some patterns in the treatment of insomnia. In our previous studies, we have observed some core drug patterns of prestigious clinicians in treatment of insomnia [15,16]. But these previous studies have some shortcomings. On the one hand, the studies are retrospective and the data were collected from daily actual clinical treatment information of prestigious clinicians. In this case, the data quality cannot be guaranteed. On the other hand, the treatment efficacy of prestigious clinicians was not validated and needed confirmation in further study. This study aims to screen the effective core drug patterns of prestigious Chinese clinicians in treatment of primary insomnia.

### Objectives

The objective of this study is to screen the effective core drug patterns in primary insomnia treatment with Chinese herbal medicine by a triple -blind, randomized, placebo-controlled trial.

## Methods

### Design

**Figure 1:**
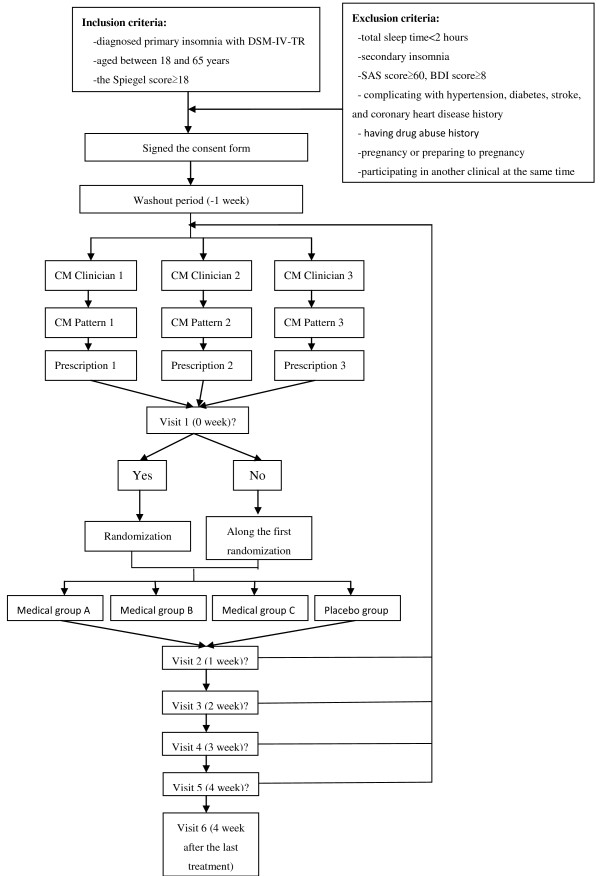
Trial design.

This is a triple-blind (with patients, clinicians, outcome assessors blinded), randomized, placebo-controlled, parallel group clinical trial. Figure 1 shows the trial design and Table 1 summarizes the timing of the trial.

**Table 1:** Calendar summary

**Period**	**Screening**	**Washout**	**Treatment**				**Follow-up**
**Week(W)**	**-1 W**	**0 W**	**1 W**	**2 W**	**3 W**	**4 W**	**4th week after the end of treatment**
Inclusion and exclusion criteria	×						
Informed consent	×						
Demography	×						
Past Medical history & treatment history	×						
Spiegel Scale	×	×					
Self-rating Anxiety Scale	×	×				×	
Beck Depression Inventory	×	×				×	
http://Pittsburgh Sleep Quality Index	×	×	×	×	×	×	×
CM syndromes and signs	×	×	×	×	×	×	×
Sleep diary	×	×	×	×	×	×	
Prescription (include every herb and dose)			×	×	×	×	
Vital signs	×	×	×	×	×	×	
Adver event		×	×	×	×	×	

### Randomization

Three Chinese clinicians will diagnose and treat every eligible patient individually. Every Chinese clinician will provide CM patterns and the prescription according to every patient’s disease condition. There will therefore be three prescriptions from three Chinese clinicians for every patient at every visit. At the first visit, patient will equally be randomized to one of the following four groups: medical group A, medical group B, medical group C or placebo group. Patients in the CM groups will be given one of three Chinese clinicians’ prescriptions. Patients in the placebo group will be given the placebo simulating the core drug patterns in the previous study. In the whole study, the randomization of group assignment only occurs at the first visit, and the randomization results will be applied to the following visits. In other words, although the patients have to be diagnosed by every clinician for every visit, they always receive the drug prescribed by the clinician who is distributed to them by randomization at the first visit or they receive placebo. Furthermore, in order to avoid the potential bias, every patient’s order to visit three clinicians is randomized at every visit, meaning that the visiting order in which one patient visits the clinicians may be different every time. It is worth mentioning that a web central randomization system is employed to guarantee the quality.

### Chinese medicine groups

After randomization, patients in the CM groups are to receive one of three Chinese clinicians’ prescriptions. Every Chinese clinician diagnoses the patients and prescribes individually based on patients’ syndrome differentiation and their own CM experiences. During the whole treatment, Chinese clinicians can make necessary adjustment in their medicinal prescription depending on the condition of the disease. The prescriptions of Chinese clinicians are pure Chinese herbs.

### Placebo group

Patients in the placebo group will receive the dummy of the core drug patterns obtained in a previous retrospective study. The previous core drug patterns include Fried semen Ziziphi Spinosae, Tuckahoe, Preparation of Polygala, Chinese Angelica root, Lotus heart, White peony root, dried tangerine peel, acorus calamus, Coptis chinensis, and Licorice health.

All CM is a herbal concentrate-granule, not a decoction. The herbal concentrate-granule, a product of high quality that can be intensively produced on a large scale, possesses many advantages, such as small dosage, easy to use and good effectiveness in clinical application. The quality of a herbal concentrate-granule can be controlled with mechanization. In China, the herbal concentrate-granule is widely accepted [17].

The placebo medicine dummy is produced by the Pharmacy Department of Guang’anmen Hospital, China Academy of Chinese Medical Sciences. The placebo is also a herbal concentrate-granule, and is similar to the true CM concentrate-granule in the aspects of color, taste, smell and package. According to the theory of CM, the prescriptions will probably be adjusted at different visits based on the patient’s disease condition. To achieve better blinding, four kinds of placebos with different tastes will therefore be designed for different visits.

One bag of herbal concentrate-granule is taken two times daily one and half hour after breakfast and dinner, respectively.

### Eligibility criteria

#### Inclusion criteria and exclusion criteria

Patients are eligible to participate if they are aged between 18 and 65 years, and meet the definition of insomnia from the *Diagnostic and Statistical Manual of Mental Disorders*, Fourth Edition, Text Revision, with Spiegel scale >18. It is imperative that patients should read and sign an informed consent form to participate in the trial.

The following exclusion criteria are observed: total sleep time <2 hours, secondary insomnia, Self-rating Anxiety Scale score >60, Beck Depression Inventory score <8, complicated with hypertension, diabetes, stroke, and coronary heart disease, having drug abuse history, pregnancy or preparing for pregnancy, and participating in another clinical trial at the same time.

### Recruitment/consent procedures

Patients are recruited by means of poster at clinic. The patient screening is conducted by the specified clinician who doesn’t participate in the study. If a patient meets the study criteria, the clinician responsible for patient screening will provide him or her with written information, explaining the study in detail in an understandable language, and obtain written consent if he or she agrees to take part in the study. All patients’ informed consent must be obtained. Any patient cannot be enrolled if she/he refuses or shows significant distress.

### The procedure of study

In the washout period, an eligible patient who has signed informed consent will receive placebo for 1 week (washout period) in order to eliminate the influence of drugs taken before the study. In the treatment period lasting 4 weeks and including four visits, each of three Chinese clinicians will independently provide a disease pattern and prescription for every patient. Three generated prescriptions will then be passed on to the pharmacy. However, whether one patient receives one of three prescriptions or placebo is dependent on the result of randomization that is conducted by the authorized person using a central randomization system at the first visit.

When a patient returns to the hospital once a week after the first treatment, a professional clinician will conduct an assessment on efficacy. The patient will then receive the secondary treatment from three Chinese clinicians, repeating the same procedure as the first one. The whole study will last 9 weeks, including 1-week washout, 4-week treatment, and follow-up happening at the 4th week after the last treatment.

All three Chinese clinicians are experts on insomnia in Prestigious Chinese Clinician Research Laboratory of Guang’anmen Hospital, China Academy of Chinese Medical Sciences. In order to minimize potential bias and to keep the blind condition of study as possible as one can, all researchers participating in the study are divided into three groups: clinical study group, diagnosis and assessment group, and quality control and statistical analysis group. In the quality control group, there are two persons (the corresponding authors) are responsible for the management and early stopping of the trial. A clinical research data capture computer system is applied to collect the data in this study.

The study was approved by the Institutional Review Board of the Institute of Basic Research in Clinical Medicine, China Academy of Chinese Medical Sciences. The approval number is 2011NO6.

### Assessment

The investigator carrying out the efficacy assessment will be blinded to which treatment the patients received. The visits and assessments will take place at screening, washout period, weeks 1, 2, 3, 4 after treatment, and the 4th week after the last treatment. All assessments will be conducted by the clinicians of the diagnosis and assessment group.

### Primary efficacy indicator

#### Total Sleep Time (TST)

In this study, the major indicator is the total sleep time (TST), which is assessed according to the sleep diary. All patients are required to record their sleeping information in detail every day. The sleep diary contains the following information: the time a patient goes to bed and gets out of bed; the time a patient thinks sleep onset occurs; the presumed cause, number, time, and length of any nighttime awakenings and activities during these moments; the name, dosage and time of any drugs used, including medication, sleep aids, caffeine and alcohol, and so forth; and activities happening 3 hours before bedtime, such as meditation, watching television, playing PC games, exercise, and so forth.

The efficacy can be confirmed only when improvement in TST is >0.5 hours and is considered statistically significant. Normally, the participants are not allowed to take any pharmacologic medicine used to treat insomnia. However, they are allowed to do so if they could not bear the bitterness brought about by insomnia. Under such circumstances, the dosage and the name of the medicine must be recorded carefully in sleep diaries.

### Secondary efficacy indicators

The secondary efficacy indicators include sleep onset latency, wake time after sleep onset, sleep efficiency, Pittsburgh sleep quality index (PSQI) and CM symptoms and signs. Sleep onset latency, wake time after sleep onset and sleep efficiency are obtained from the sleep diary. The Chinese-version PSQI is employed to measure the quality of sleep. The PSQI is a self-rated questionnaire used to assess sleep quality and disturbances over a 1-month time interval. Nineteen individual items generate seven component scores: subjective sleep quality, sleep latency, sleep duration, habitual sleep efficiency, sleep disturbances, use of sleeping medication, and daytime dysfunction.

Many studies have demonstrated that the Chinese-version PSQI is a sensitive, reliable, and valid tool to assess the quality of sleep [18,19]. In addition, CM symptoms and signs will be assessed. The main symptoms and signs include headache, dizziness, spontaneous sweating, night sweating, dreaminess, amnesia, anorexia, lassitude of spirit and lack of strength, oppression in the chest, daytime sleepiness, irritability, palpitations, epigastric fullness, tinnitus, thirst, bitter taste in the mouth, abundant sputum, yellow urine, dry stool, sloppy stool, pulse condition, and tongue manifestation.

### Safety

Safety assessments will be based on adverse event reports and vital signs. At every visit, adverse events and vital signs will be recorded. The major indicators for vital signs include breath, temperature, systolic blood pressure, diastolic pressure, pulse, and so on. Generally, any unexpected symptom, vital sign or sickness, as long as they cause discomfort, shall be recorded as an adverse event. The starting date, the ending date, the degree, the relations with the trial medicine, and whether they drop out of the study should be recorded correspondingly. If necessary, the patients will receive relevant treatment. If the adverse event still exists, the follow-up will go on until the adverse event disappears.

### Statistical methods

#### Sample size

The study is supposed to explore the daily clinical practice of three prestigious Chinese clinicians in primary insomnia treatment and to figure out the effective drug patterns prescribed by them. However, it is very difficult to estimate the sample size because of lack of a previous relevant study. To obtain expected results with a minimal sample size, a sequential design will be employed. The interim analysis will be conducted when the total number of patients having completed the study reaches 80 and 160 separately, for which we set up the early stopping criteria. The interim study can be treated as final if the difference in TST changes from baseline at 4 weeks between the CM and placebo groups is >0.5 hours and has statistical significance with *P* <0.05. When the early stopping criteria are met, the placebo group and the non-effective group will be dropped. The subsequent study will only focus on the effective group to explore the core drug patterns. If the number of effective patients is not sufficient to determine the core drug patterns, the recruitment procedure has to continue. The core drug pattern analysis shall be carried out every time the increment of new enrollment reaches 30 until the core drug patterns are obtained and kept constant.

### Analysis strategy

The analysis will be per intent to treat. The mean and standard deviation shall be applied to the continuous variables, and percentages to the categorical variables. For comparison of two independent samples, the *t* test and analysis of variance will be applied for continuous variables and the chi-square test for categorical variables. In addition, nonparametric tests will also be applied for analysis. The efficacy for patients taking any pharmacologic medicine will be assessed using the method of last observation carried forward.

As mentioned above, the exploration analysis on core drug patterns will only be based on the effective patients in order to guarantee the efficacy of the explored core prescription. The patients’ syndrome and signs, CM patterns and the treatment effect shall be considered during the analysis. The core drug pattern can be concluded from the combination of high correlation and frequency. The core drug patterns must confirm to the CM theory and be accepted by the prestigious Chinese clinicians. The multi-dimension association rule and scale-free networks method will be applied to mine the core drug patterns and drug utilization rule of three Chinese clinicians [15,20-22]. In CM, the disease syndromes and signs, disease pattern and treatment are a dynamic procedure. The disease pattern varies with syndromes and signs. As a result, the treatment varies with the pattern of disease. So we not only explore the core drug patterns of different prestigious Chinese clinicians, but also mine the core drug patterns corresponding to different syndrome combinations and disease patterns.

## Discussion

The discovery of a new effective prescription in clinical practice is key to the development of new CM. The experiences of prestigious Chinese clinicians are the main source of effective CM. However, the discovery of effective core drug patterns mainly depends on the accumulation of clinicians’ experiences and is always a long process. This study proposes one method used to develop new effective core drug patterns based on the prestigious clinicians’ treatment data. Randomization, placebo control and blinding designs are employed in the study, where three prestigious Chinese clinicians are treated as subjects. To minimize the bias and factitious influence, a web central randomization system and a clinical research data-capture computer system are adopted to assign the group and collect the data, respectively. In the study, we can obtain one or several effective core drug patterns. Unlike previous retrospective studies [15,16], this perspective study has strictly tested the efficacy of Chinese herbal medicine through comparison between a treatment group and a placebo group. Because the exploratory analysis of core drug patterns is based on the effective patients, the results should be more accurate and useful. In a word, the analysis can accelerate and improve the development of new Chinese herbal medicine for discovery of new effective core drug patterns through exploring the treatment data of prestigious CM clinicians.

To guarantee the quality of the study and accuracy of core herb patterns obtained, patients who met the diagnosis of primary insomnia were recruited. In addition, those patients with hypertension, diabetes, stroke and coronary heart diseases are excluded because these patients may demonstrate mixed symptoms or syndromes in the diagnosis that affect the accuracy of prescriptions by clinicians. Patients older than 65 are easily complicated with the diseases mentioned above and then excluded from the study.

In Chinese medical treatment, different Chinese clinicians possess different ideas on diagnosis and treatment. One or more than one core prescriptions could therefore possibly be obtained. In this case, the question is raised about whether the explored core prescription can be generalized from this relatively small group. On one hand, the three Chinese clinicians are experts in this field and can represent the best in China. As a result, their prescriptions could be treated as valuable. On the other hand, in the study the clinicians are actually considered as treatment instead of as sample. Hence, there should not be a problem of small size for clinicians. Nevertheless, further randomized controlled trial study will be done to examine the efficacy of the core prescription.

There is another factor that needs to be explained – that the primary outcome TST is from a sleep diary but not from an objective measure (polysomnographic or actigraphy). TST from a sleep diary by patient self-reporting has been efficiently used in most of the treatment studies on insomnia. Although it is subjective and there is often a divergence compared with objective sleep measures, we think it is acceptable because our study is an exploratory study and in clinical practice many patients with insomnia do not receive overnight sleep monitoring.

## Abbreviations

CM: Chinese medicine; DSM-IV-TR: Diagnostic and Statistical Manual of Mental Disorders, Fourth Edition, Text Revision; SAS: Self-rating Anxiety Scale; BDI: Beck Depression Inventory; TST: total sleep time; PSQI: Pittsburgh Sleep Quality Index; AE: Adverse Event; ITT: Intent to Treat.

## Competing interests

The authors declare that they have no competing interests.

## Authors’ contributions

SY participated in the design of the study, provided statistical advice and drafted the manuscript. RZ was in charge of the placebo preparation, the randomization and coordination development of the trial. XZ participated in the design of the study and provided statistical advice. PL was in charge of the recruitment of patients, data collection and management. LH and BL conceived the study, participated in its design and coordinated development of the trial. All the authors read and approved the final manuscript.
